# Monopolar teres major muscle transposition to improve shoulder abduction and flexion in children with sequelae of obstetric brachial plexus palsy

**DOI:** 10.1186/1749-7221-4-20

**Published:** 2009-10-26

**Authors:** Jörg Bahm, Claudia Ocampo-Pavez

**Affiliations:** 1 Euregio Reconstructive Microsurgery Unit, Franziskushospital, Aachen, Germany

## Abstract

We present a new surgical technique for a pedicled teres major muscle transfer to improve shoulder abduction and flexion in children with sequelae of obstetric brachial plexus palsy.

In addition, we provide the clinical outcome in the first 17 operated children.

## Introduction

Muscle weakness is a frequent sequela after obstetric brachial plexus palsy (obpp) and might be improved by muscle transpositions, especially at the shoulder level [1]. The teres major muscle (tmm) is included in the technique described by Hoffer [2] to enhance active lateral rotation of the shoulder, where this muscle should address the function of the infraspinatus muscle.

We propose a single transfer of the tmm in selected conditions in children suffering obpp sequelae:

1. when shoulder flexion and/or abduction are weak against gravity (active ROM less than 90° with a strength less or equal M3)

2. when the tmm shows cocontractions during shoulder abduction (mixed reinnervation of the dorsal cord)

3. to add muscle volume to a cranial trapezius transfer for weak shoulder abduction

4. to modify a Hoffer transfer [2], using the latissimus dorsi muscle (ldm) to improve the lateral shoulder rotation with an abducted arm, and tmm to allow an active abduction up to 90° (horizontal line), which will bring the transferred ldm under good tension.

Essentially, the tmm might be considered as a valuable functional muscle transfer to enhance shoulder abduction and elevation in selected children with obpp sequelae, under 10 years of age with reasonable body weight. The muscle thereby improves prime movers of the shoulder joint.

### Surgical Technique (figure 1)

**Figure 1:**
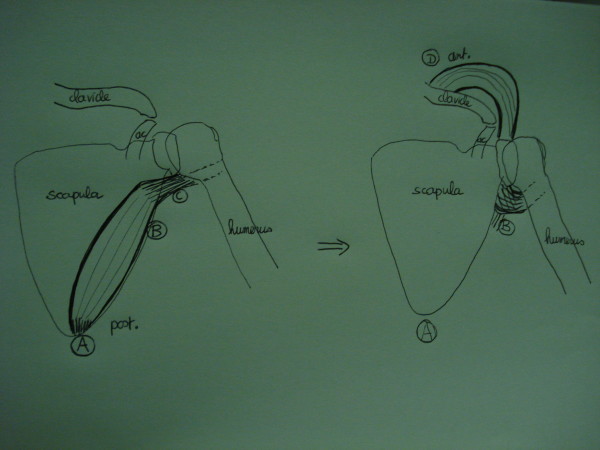
**Scheme explaining the harvest and transposition of the teres major muscle**. A: detachment of all distal insertions of the tmm onto the lower scapular angle. B: pivot point at the level of the neurovascular bundle. C: tunnel under and medial to the proximal humerus, beside the maintained conjoint tendon insertion. D: new fixation onto the clavicle or deltoid muscle attachment.

The child is placed in a lateral position under general anesthesia. A double access is needed to the midaxillar line (to detach the muscle) and to the acromio-clavicular region (to transpose the muscle onto the antero-lateral deltoid muscle (dm) insertion).

**Figure 2:**
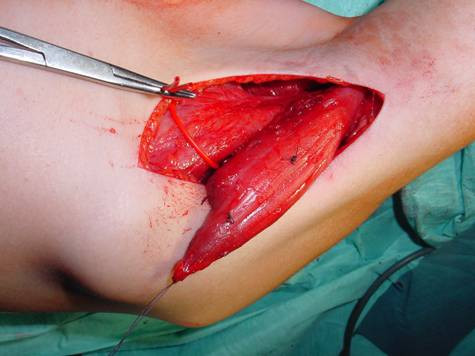
**Harvest of the tmm detached from the inferior scapular angle**.

A strait skin incision is drawn beginning in the axilla following down the midaxillar line until the lower angle of the scapula. The subcutaneous tissue is divided, and the lateral borders of both ldm and tmm are identified and dissected free. The tmm is dissected free from the ldm progressively from its lateral border, from proximal maintaining its tendon insertion onto the humerus down to the lower scapular angle, where it is completely detached. The medial border is freed from distal to proximal; and particular attention is paid to preserve the neurovascular bundle, which lies at the deeper proximal lateral border, a few cm above the well visible bundle to the ldm (figure 2).

The dissection continues until the tmm is freed all around and maintains only its proximal tendon and the neurovascular bundle. At this stage, the free muscular rim may be reinforced by several absorbable mattress sutures, or a running suture, with a long suture end which will be grasped to further mobilize the muscle.

A second incision is conducted from the proximal delto-pectoral groove about 5 cm more proximally; the subcutaneous fat is divided and the cephalic vein is respected; the often hypotrophic anterior and middle parts of the dm are identified and their insertion on the lateral clavicle dissected free. From this approach, using the upper delto-pectoral access, a tunnel is prepared, going under the dm, more laterally and distally, crossing above the humerus. From the other incision, in line with the respected conjoined tendon, the tunnel is completed moving over the humerus, to join the dissecting finger(s) from above.

**Figure 3:**
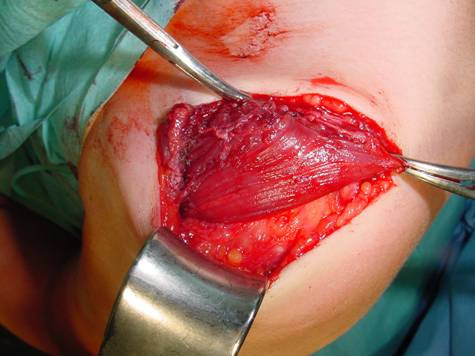
**The muscle is positioned to replace/augment the anterior and lateral part of the deltoid muscle**.

The tunnel is widened for 2 fingers by gentle blunt dissection and after myorelaxation has been obtained (curarisation by the anaesthesiologist), the distal end of the tmm is passed through the tunnel (figure 3).

The midaxillar incision is closed over a little drain; the muscle is inserted unto the lateral clavicular rim unto the remaining dm with the arm positioned in 90° abduction and 20° flexion. This fixation is realized by several Maxon 2/0 sutures passed behind the rim suture on the tmm, so that a tight connection to the dm insertion unto the clavicle might be obtained.

An abduction orthesis is maintained for 6 weeks and than progressive active mobilisation is performed.

## Results

In a continous series from July 2005 to March 2009, we performed the tmm transfer in 17 children aged 3 to 17 years, and obtained improvements both in shoulder abduction (between 15 and 70°) and flexion (50°) after a follow-up ranging from 5 to 36 months.

One muscle was lost probably by injury to the neurovascular bundle in a rather fibrotic muscle with difficult dissection; the completely necrotized muscle had to be withdrawn after 1 week. There were no other drawbacks.

## Discussion

We believe that the tmm transfer, based on its unique vascular pedicle (a branch of the subscapular artery) and nerve (a direct motor branch from the posterior cord) as a monopedicular transfer (maintaining the proximal tendon insertion), is functionally an interesting option to enhance muscle strength, and to counteract co-contractions at the shoulder level in children with obpp sequelae.

This transfer might also be used to enhance the muscle bulk in a cranial trapezius muscle transfer or in a modified Hoffer transfer for lateral rotation of the shoulder.

The critical point of the surgery is the identification of the unique neurovascular bundle and the transposition through a previously widened tunnel over the humerus, and under the remaining dm, without compromising the muscle viability.

Our good preliminary functional results encourage us to further develop and advise this transposition technique.

## Summary

We present a new surgical technique, using the monopolar teres major muscle transfer to enhance shoulder function in children suffering from sequelae of upper obstetric brachial plexus palsy.

## Competing interests

The authors declare that they have no competing interests.

## Authors' contributions

JB developed the technique and wrote the manuscript; COP participated in the surgeries and in the clinical follow-up of patients. Both authors read and approved the final version of the manuscript.
